# Supplementary material for: An atopic dermatitis-like murine model by skin-brushed cockroach Per a 2 and oral tolerance induction by Lactococcus lactis-derived Per a 2
Source: PLoS One. 2023 Sep 7;18(9):e0291162. doi: 10.1371/journal.pone.0291162 (PMC10484430; doi:10.1371/journal.pone.0291162)
Supplement: S1 Table — (DOCX) [file pone.0291162.s001.docx]

**Supplementary Table 1.**

**The sequences of murine gene-specific primers used in real-time PCR**

| **Gene (mouse)** | | **Sequences** | **Produce size (bp)** |
| --- | --- | --- | --- |
| ***Il4*** | F | 5’ AGC CAT ATC CAC GGA TGC GAC AAA 3’ | 176 |
|  | R | 5’ AAT ATG CGA AGC ACC TTG GAA GCC 3’ |  |
| ***Il13*** | F | 5’ AGA CCA GAC TCC CCT GTG CA 3’ | 123 |
|  | R | 5’ TGG GTC CTG TAG ATG GCA TTG 3’ |  |
| ***Ifnγ*** | F | 5’ GGC CAT CAG CAA CAA CAT AAG CGT 3’ | 118 |
|  | R | 5’ TGG GTT GTT GAC CTC AAA CTT GGC 3’ |  |
| ***Il31*** | F | 5’ CAG CTG TTT CAA CCC ACT G 3’ | 121 |
|  | R | 5’ CAG TTC TGC CAT GCA GTT TG 3’ |  |
| ***Il33*** | F | 5’ ATTTCCCCGGCAAAGTTCAG3’ | 118 |
|  | R | 5’AACGGAGTCTCATGCAGTAGA3’ |  |
| ***Tslp*** | F | 5’-GGA GAT TTG AAA GGG GCT AAG -3’ | 168 |
|  | R | 5’- TGG GCA GTG GTC ATT GAG- 3’ |  |
| ***Actb*** | F | 5’ GGC CAA CCG TGA AAA GAT GA 3’ | 251 |
|  | R | 5’ CAC GCT CGG TCA GGA TCT TC 3’ |  |
