# Supplementary material for: An atopic dermatitis-like murine model by skin-brushed cockroach Per a 2 and oral tolerance induction by Lactococcus lactis-derived Per a 2
Source: PLoS One. 2023 Sep 7;18(9):e0291162. doi: 10.1371/journal.pone.0291162 (PMC10484430; doi:10.1371/journal.pone.0291162)
Supplement: Attachment — Submitted filename: My response.docx [file pone.0291162.s003.docx]

Dear Editor,

The investigative group of the manuscript “An atopic dermatitis-like murine model by skin-brushed cockroach Per a 2 and oral tolerance induction by Lactococcus lactis-derived Per a 2” have satisfactorily answered all queries and now ready to publish.

Thanks
